# The association between severe menopausal symptoms and engagement with HIV care and treatment in women living with HIV

**DOI:** 10.1080/09540121.2020.1748559

**Published:** 2020-04-11

**Authors:** Danielle Solomon, Caroline A. Sabin, Fiona Burns, Richard Gilson, Sris Allan, Annamiek de Ruiter, Rageshri Dhairyawan, Julie Fox, Yvonne Gilleece, Rachael Jones, Frank Post, Iain Reeves, Jonathan Ross, Andrew Ustianowski, Jane Shepherd, S. Tariq

**Affiliations:** a Institute for Global Health, University College London, UK; b Royal Free London NHS Foundation Trust, UK; c Coventry and Warwickshire Partnership NHS Trust, UK; d Guy’s & St Thomas’ NHS Foundation Trust, UK; e ViiV Healthcare, UK; f Barking, Havering and Redbridge NHS Trust, UK; g Brighton & Sussex University Hospitals NHS Trust, UK; h Brighton & Sussex Medical School, UK; i Chelsea and Westminster Hospital NHS Foundation Trust, UK; j King's College Hospital NHS Foundation Trust, UK; k Homerton University Hospital NHS Foundation Trust, UK; l University Hospital Birmingham NHS Foundation Trust, UK; m North Manchester General Hospital, Penine Acute Hospitals NHS Trust, UK; n UK Community Advisory Board

**Keywords:** Menopause, ageing, women, hiv, adherence, clinic attendance

## Abstract

Using data from the PRIME Study, an observational study of the menopause in women living with HIV in England, we explored the association between menopausal symptoms and: (i) antiretroviral therapy (ART) adherence and (ii) HIV clinic attendance.

We measured menopausal symptom severity with the Menopause Rating Scale (MRS, score ≥17 indicating severe symptoms), adherence with the CPCRA Antiretroviral Medication Adherence Self-Report Form, and ascertained HIV clinic attendance via self-report. Odds ratios were obtained using logistic regression.

Women who reported severe menopausal symptoms had greater odds of suboptimal ART adherence (adjusted odds ratio (AOR) 2.22; 95% CI 1.13, 4.35) and suboptimal clinic attendance (AOR 1.52; 95% CI 1.01, 2.29). When psychological, somatic and urogenital domains of the MRS were analysed individually there was no association between adherence and severe symptoms (all *p* > 0.1), however there was an association between suboptimal HIV clinic attendance and severe somatic (AOR 1.98; 95% CI 1.24, 3.16) and psychological (AOR 1.76; 95% CI 1.17, 2.65) symptoms.

Severe menopausal symptoms were significantly associated with sub-optimal ART adherence and HIV clinic attendance, however we cannot infer causality, highlighting the need for longitudinal data.

## Introduction

In 2017, over half of the estimated 34.5 million adults living with HIV globally were female (UNAIDS Data, 2017, 2017); in the United Kingdom, there were an estimated 31,600 women living with HIV in 2015 (Kirwan PD et al., 2016), nearly a third of all those living with HIV nationally. As antiretroviral therapy (ART) has become more effective and more widely available, HIV has transformed from a life-limiting condition into one, for those who are well-controlled on treatment, with near-normal life expectancy (Samji et al., 2013). This has resulted in increasing numbers of people living with HIV surviving into older age, meaning that effective HIV management requires an understanding of the physiological and psychological processes of ageing.

For women, an important aspect of ageing is the menopause. In 2018, approximately 11,100 (Khawam, 2019) women of potentially menopausal age (45–56 years) attended for HIV care in the UK, a 5-fold increase over 10 years. Although the data are conflicting, several studies have indicated that women with HIV may experience menopause at an earlier age than their HIV-negative counterparts (Tariq et al., 2016). In addition, women with HIV experience a high rate of vasomotor, urogenital and psychological menopause-related symptoms – higher, in some studies, than among women who are not living with HIV (Ferreira et al., 2007; Looby et al., 2014; Looby et al., 2018) - while simultaneously reporting low rates of hormone replacement therapy (HRT) use (Clark et al., 2000; Samuel et al., 2014; Tariq S et al., 2017).

Despite the clinical need, there is currently a paucity of data on the impact of the menopause transition on women’s engagement in HIV care, with only one study to date examining the association between menopausal symptoms and ART adherence (Duff et al., 2018). Suboptimal ART adherence and poor HIV clinic attendance have a significant impact on clinical outcomes and have been associated with reduced rates of virological suppression, which may lead to increased HIV-related morbidity and onward transmission of HIV (Apisarnthanarak & Mundy, 2010; Bastard et al., 2012).

We hypothesise that menopausal symptoms negatively impact women’s capacity to engage with HIV care and treatment. Using quantitative data from the PRIME Study, an observational study of the menopause in women living with HIV in England, we aim to explore the association between the menopause transition and engagement in HIV care. Our specific objectives are to examine the association between menopausal status and symptoms with (i) ART adherence and (ii) HIV clinic attendance.

## Methods

We undertook an analysis of quantitative data from women living with HIV aged 45–60 in England who participated in the PRIME (Positive TRansItions through the MEnopause) Study.

### The PRIME study

The PRIME study is a mixed-methods observational study exploring the impact of the menopause on the health and well-being of women living with HIV. The study recruited 869 women aged 45–60 attending one of 21 National Health Service (NHS) HIV clinics in England between February 2016 and June 2017. Participants completed self-administered paper-based questionnaires (including validated questions on menopausal status and symptomatology), with a sub-sample taking part in semi-structured qualitative interviews (n = 20). This was supplemented with routine clinical data. Methods are described in detail elsewhere (Tariq et al., 2019).

### Variables

We ascertained menopausal status through self-reported menstrual pattern (Brambilla et al., 1994); categorising women as premenopausal (reporting regular menstruation), perimenopausal (reporting irregular periods over the previous two years), and postmenopausal (amenorrhoea for twelve months or more) (Brambilla et al., 1994). The Menopause Rating Scale (MRS) was used to ascertain presence and severity of menopausal symptoms; MRS has been validated, and has an internal consistency coefficient (Cronbach’s alpha) of 0.83 (Heinemann et al., 2004). The MRS comprises 11 items (symptoms or complaints) across three domains: psychological (e.g., Depression and anxiety), somatic (e.g., Vasomotor symptoms and sleep disturbance) and urogenital (e.g., Vaginal dryness and sexual problems). Each of the eleven symptoms are rated on a Likert scale between 0 (no symptoms) and 4 (severe symptoms) by participants. Menopausal symptoms among PRIME participants were analysed within each of the three domains, and also as a composite measure. For this analysis, a score ≥7 in the psychological domain indicated severe psychological symptoms, a score ≥9 in the somatic domain indicated severe somatic symptoms, a score ≥4 in the urogenital domain indicated severe urogenital symptoms, and a cumulative total score ≥17 indicated severe menopausal symptoms as a composite (Berlin Center for Epidemiology and Health Research).

ART adherence was measured using the validated Terry Beirn Community Programs for Clinical Research on AIDS (CPCRA) Antiretroviral Medication Adherence Self-Report Form (O'Connor et al., 2016). Participants were asked to complete the following: “during the last 7 days I took” and selecting one of the following options: “all”, “most”, “about one-half”, “very few”, or “none” “of my pills”. This was dichotomised into optimal (100% adherence in the past 7 days) or suboptimal (<100% adherence in the past 7 days) adherence.

HIV clinic attendance was assessed using the question: “in the past 12 months, have you missed any appointments at this clinic?” Responses were dichotomised into complete (100%) or incomplete (<100%) attendance in the past 12 months.

### Statistical analysis

We present percentages with associated 95% confidence intervals (CI), as well as medians and interquartile ranges (IQR). Chi-squared tests were used to identify statistically significant associations (*p *< 0.05) between menopausal status and menopausal symptoms (as separate domains, and as a composite measure) and (i) ART adherence and (ii) HIV clinic attendance.

We identified ethnicity *a priori* as a confounding variable from the literature (Green & Santoro, 2009; Simoni et al., 2012). We performed chi-squared tests to assess the relationship between demographic and HIV-related factors, and the dependent and independent variables, and factors with association of *p *< 0.2 with *both* likelihood of severe menopausal symptoms *and* likelihood of suboptimal ART adherence or HIV clinic attendance were included in multivariable models. Using these metrics, ethnicity, employment, high-risk alcohol use, current smoking, basic needs met and years since diagnosis were added to the adherence model, and age and ethnicity were added to the attendance model.

We used multivariable logistic regression models to calculate odds ratios (ORs) for reporting either suboptimal ART adherence or suboptimal HIV clinic attendance given a woman’s menopausal status and severity of symptoms, adjusting for potential confounders. Similar models were used to calculate ORs for the relationship between the number of domains (psychological, urogenital and/or somatic) in which a woman reported severe menopausal symptoms, and suboptimal adherence or clinic attendance.

All statistical analyses were performed using Stata 15 (StataCorp. 2017. Stata Statistical Software: Release 15. College Station, TX: StataCorp LLC).

### Ethics

The PRIME Study has ethical approval from the South East Coast-Surrey Research Ethics Committee (REF 15/0735).

## Results

The sample was restricted to the 701 women with available data on menopausal status and symptoms (missing for 168/869). The adherence analysis included women who reported being on ART and had available data on their adherence (661/869), while the clinic attendance analysis included women who had available data on their HIV clinic attendance, regardless of whether or not they were on ART (692/869).

### Adherence to ART

The demographic breakdown of the adherence sample was very similar to that of the entire PRIME dataset (Table 1). The 661 women included in the analyses had a median age of 49 (IQR 47–52) years (Table 1). Over two-thirds were of Black African ethnicity (72.6%). The majority of women were in either full- or part-time employment (69.3%), and just under half had attended university (47.0%). Approximately two-thirds of the sample (65.4%) reported having enough money to meet their basic needs most or all of the time. There were low rates of smoking (8.7%), high risk alcohol use (AUDIT- C ≥5, 9.0%) and recreational drug use (2.6%). The median number of years since HIV diagnosis was 14 (IQR 10–18). The majority of women had an undetectable HIV viral load (89.0%) with 69.3% having a CD4 count ≥500 cells/mm^3^ when last tested. Ten percent (n = 65) of women in our sample reported suboptimal ART adherence within the past seven days.

**Table 1.** Univariate descriptive and χ2 analysis of associations between patient characteristics and engagement with care.

	PRIME sample group	Adherence analysis		Optimal adherence	Suboptimal adherence	*p* value^a^		Attendance analysis	Optimal attendance	Suboptimal attendance	*p* value^a^
	N = 869 (%)	N = 661 (%)		N = 596 (91.2%)	N = 65 (9.8%)			N = 692 (%)	N = 549 (80.3%)	N = 143 (20.7%)	
Median age in years, (range)	49 (47–52.5)	49 (47–52)		49 (47–52)	49 (45–51)	0.756		49 (45–51)	50 (45–52)	49 (46–52)	0.09
Ethnicity						0.094					0.12
Black African	607 (72.2)	472 (72.6)		430 (73.5)	42 (64.6)			487 (71.5)	381 (70.7)	106 (74.7)	
White UK	71 (8.4)	59 (9.1)		48 (8.2)	11 (16.9)			62 (9.1)	53 (9.8)	9 (6.3)	
Other	163 (19.4)	132 (18.3)		107 (18.3)	12 (18.5)			132 (19.4)	105 (19.5)	27 (19.0)	
Employment						<0.001					0.35
Employed	552 (66.3)	442 (69.3)		409 (71.1)	33 (52.4)			465 (69.6)	377 (70.7)	88 (65.2)	
Unemployed	281 (33.7)	196 (30.7)		166 (28.9)	30 (47.6)			203 (30.4)	156 (29.3)	47 (34.8)	
Education						0.15					0.64
Did not complete school	93 (11.5)	68 (10.7)		62 (10.8)	6 (9.7)			70 (10.5)	52 (9.9)	18 (12.9)	
“O” level^b^	188 (23.2)	143 (22.6)		132 (23.1)	11 (17.7)			153 (23.0)	125 (23.9)	28 (20.0)	
“A” level^c^	170 (21.0)	125 (19.7)		106 (18.5)	19 (30.7)			135 (20.3)	107 (20.4)	28 (20.0)	
University	360 (44.4)	298 (47.0)		272 (47.6)	26 (41.9)			306 (46.1)	240 (45.8)	66 (47.1)	
Enough money for basic needs						0.013					0.14
All the time	312 (36.5)	262 (39.9)		248 (41.9)	14 (21.9)			277 (40.3)	226 (41.5)	51 (35.9)	
Most of the time	225 (26.3)	167 (25.5)		143 (24.2)	24 (37.5)			170 (24.8)	140 (25.7)	30 (21.1)	
Some/None of the time	319 (37.3)	227 (34.6)		201 (33.9)	26 (40.6)			240 (34.9)	179 (32.8)	61 (43.0)	
Smoking						0.003					0.8
No	776 (91.7)	590 (91.3)		537 (92.4)	53 (81.5)			618 (91.4)	492 (91.2)	126 (92.0)	
Yes	70 (8.3)	56 (8.7)		44 (7.6)	12 (18.5)			58 (8.6)	47 (8.7)	11 (8.0)	
High risk alcohol use^d^						<0.001					0.85
No	736 (91.4)	567 (91.0)		522 (92.7)	45 (75.0)			595 (91.0)	470 (91.1)	125 (90.6)	
Yes	69 (8.6)	56 (9.0)		41 (7.3)	15 (25.0)			59 (9.0)	46 (8.9)	13 (9.4)	
Recreational drug use						0.762					0.51
No	824 (97.5)	627 (97.4)		567 (97.4)	60 (96.8)			655 (97.2)	523 (97.4)	132 (96.3)	
Yes	21 (2.5)	17 (2.6)		15 (2.6)	2 (3.2)			19 (2.8)	14 (2.6)	5 (3.7)	
Median years since diagnosis (interquartile range)	14 (9–18)	14 (10–18)		14 (9–18)	16 (12.5–22)	0.005		14 (10–18)	14 (10–18)	13 (9–18)	0.89
Most recent CD4 count (cells/mm^3^)						0.383					0.47
≥500	519 (68.2)	409 (69.3)		363 (68.6)	46 (75.4)			428 (69.5)	347 (70.5)	81 (65.3)	
200–499	192 (25.2)	139 (23.6)		126 (23.8)	13 (21.3)			145 (23.5)	113 (23.0)	32 (25.8)	
<200	50 (6.6)	42 (7.1)		40 (7.6)	2 (3.3)			43 (7.0)	32 (6.5)	11 (8.8)	
Most recent HIV viral load						0.676					
Undetectable	717 (88.0)	561 (89.0)		505 (89.2)	56 (87.5)			578 (87.8)	465 (89.1)	113 (83.9)	0.057
Detectable	98 (12.0)	69 (11.0)		61 (10.8)	8 (12.5)			80 (12.2)	57 (10.1)	23 (16.1)	

	Adherence analysis		Optimal adherence	Suboptimal adherence	*p* value^a^		Attendance analysis	Optimal attendance	Suboptimal attendance	
	N = 661 (%)		N = 596 (91.2%)	N = 65 (9.8%)			N = 692 (%)	N = 549 (80.3%)	N = 143 (20.7%)	*p* value^a^
Menopausal status					0.897					0.73
Pre-menopausal	146 (22.1)		132 (22.2)	14 (21.5)			151 (21.8)	119 (21.7)	32 (22.4)	
Peri-menopausal	298 (45.1)		270 (45.3)	28 (43.1)			309 (44.7)	242 (44.1)	67 (46.9)	
Post-menopausal	217 (32.8)		194 (32.6)	23 (35.4)			232 (33.5)	188 (34.2)	44 (30.8)	
All menopausal symptoms					<0.001					0.14
None/mild/moderate	475 (71.9)		445 (74.7)	30 (46.2)			499 (72.1)	403 (73.4)	96 (67.1)	
Severe	186 (28.1)		155 (25.3)	35 (53.9)			193 (27.9)	146 (26.6)	47 (32.9)	
Somatic menopausal symptoms										
None/mild/moderate	555 (84.0)		504 (84.6)	51 (78.5)	0.203		581 (84.0)	471 (81.1)	78 (70.3)	0.01
Severe	106 (16.0)		92 (15.4)	14 (21.5)			111 (16.0)	110 (18.9)	33 (29.7)	
Psychological menopausal symptoms										
None/mild/moderate	476 (72.0)		442 (74.2)	34 (52.3)	<0.001		501 (72.4)	409 (81.6)	140 (73.3)	0.02
Severe	185 (28.0)		154 (25.8)	31 (47.7)			191 (27.6)	92 (18.4)	51 (26.7)	
Urogenital menopausal symptoms										
None/mild/moderate	464 (70.2)		424 (71.1)	40 (61.5)	0.108		490 (70.8)	389 (79.4)	160 (79.2)	0.96
Severe	197 (29.8)		172 (28.9)	25 (38.5)			202 (29.2)	101 (20.6)	42 (20.8)	

^a^ χ^2^ or Kruskal-Wallis test;^b^ equivalent to completing US Grade 10; ^c^ equivalent to completing US Grade 12; ^d^ using the Alcohol Use Disorders Identification Test (AUDIT-C) screening tool.

The majority of women were perimenopausal (45.1%), with 32.8% being postmenopausal and 22.8% being premenopausal. Over a quarter (28.0%) reported severe psychological symptoms, nearly a third reported severe urogenital symptoms (29.8%) and 16.0% reported severe somatic symptoms. Over a quarter (28.1%) had a cumulative MRS score ≥17, indicating that they were experiencing severe menopausal symptoms as a composite measure. On χ^2^ analysis (Table 1), suboptimal ART adherence was associated with being unemployed, not having enough money to meet basic needs, being a current smoker, high-risk alcohol use, and longer duration of diagnosis (all *p *< 0.05). It was not associated with age or level of education. Over half of women reporting suboptimal ART adherence also reported severe menopausal symptoms, compared to 25.3% of those reporting optimal adherence (*p *< 0.001). Menopausal status (pre-, peri- or postmenopausal) was not associated with suboptimal adherence.

Women with severe menopausal symptoms had over three times the odds of suboptimal ART adherence compared to those without (OR 3.44; 95% CI 2.04, 5.79). When adjusting for ethnicity, employment, high-risk alcohol use, current smoking, basic needs met and years since diagnosis, the association was attenuated but remained statistically significant (adjusted odds ratio (AOR) 2.22; 95% CI 1.13, 4.35, Table 2).

**Table 2.** Univariable and multivariable logistic regression analyses of the association between menopausal symptoms and suboptimal adherence to ART.

		**Suboptimal adherence to ART**	
		OR	95% CI
**All menopausal symptoms**	None/mild/moderate (MRS <17)	**Ref**	
			
	Severe (MRS ≥17)	**3.44**	**2.04**–**5.79**
Multivariable analysis (all symptoms)*	None/mild/moderate	**Ref**	
	Severe	**2.22**	**1.13–4.35**
**Somatic menopausal symptoms**	None/mild/moderate	Ref	
	Severe	1.50	0.80–2.83
Multivariable analysis (somatic)*	None/mild/moderate	Ref	
	Severe	0.59	0.27–1.28
**Psychological menopausal symptoms**	None/mild/moderate	**Ref**	
	Severe	**2.62**	**1.56**–**4.40**
Multivariable analysis (psychological)*	None/mild/moderate	Ref	
	Severe	1.36	0.71–2.64
**Urogenital menopausal symptoms**	None/mild/moderate	Ref	
	Severe	1.54	0.91–2.62
Multivariable analysis (urogenital)*	None/mild/moderate	Ref	
	Severe	0.96	0.51–1.81
**Number of severe symptoms**	0/1	Ref	
	2/3	**2.01**	**1.16–3.49**
Multivariable analysis (number of severe symptoms)*	0/1	Ref	
	2/3	0.90	0.45–1.79
**Menopausal status**	Premenopausal	Ref	
	Perimenopausal	0.98	0.50–1.92
	Postmenopausal	1.12	0.55–2.25

* adjusted for ethnicity, employment, high-risk alcohol use, current smoking, basic needs met and years since diagnosis

As the MRS score is divided into three sub-domains, it is possible to exhibit severe symptoms in one domain without having a severe cumulative MRS score. We therefore conducted a logistic regression analysis of the relationship between adherence and MRS sub-domains. We found no association between suboptimal ART adherence and severe somatic (AOR 0.59; 95% CI 0.27, 1.28), psychological (AOR 1.36; 95% CI 0.71, 2.64) or urogenital symptoms (AOR 0.96; 95% CI 0.51, 1.81). Women experiencing severe symptoms in two or three domains had over twice the odds of suboptimal ART adherence when compared to women who reported no severe symptoms or one domain only (OR 2.01; 95% CI 1.16–3.49), however this association did not persist on multivariable analysis (AOR 0.90; 95% CI 0.45– 1.79, Table 2). The attenuation of this association was predominantly mediated by adjustment for employment status.

### Clinic attendance

The baseline characteristics of the HIV clinic attendance sample were similar to those of the adherence sample (Table 1).

A detectable HIV viral load was present in a greater proportion of women who reported missing ≥1 HIV clinic appointment compared to those who had not missed any (16.1% vs. 10.1%, *p* = 0.057, Table 1). We did not find an association between suboptimal HIV clinic attendance and age, education, ethnicity, employment, smoking, income, alcohol consumption, last CD4 count or menopausal status (all *p *> 0.08, Table 1).

Women with severe menopausal symptoms had greater odds of suboptimal HIV clinic attendance than those without, adjusting for ethnicity and age (AOR 1.52; 95% CI 1.01, 2.29; Table 3).

**Table 3.** Univariable and multivariable logistic regression analyses of the association between menopausal symptoms and suboptimal HIV clinic attendance.

		**Suboptimal HIV clinic attendance**	
		OR	95% CI
**All menopausal symptoms**	None/mild/moderate (MRS <17)	Ref	
			
	Severe (MRS ≥17)	1.35	0.91–2.01
			
Multivariable analysis (all symptoms)*	None/mild/moderate	Ref	
	Severe	1.52	1.01–2.29
**Somatic menopausal symptoms**	None/mild/moderate	**Ref**	
	Severe	**1.81**	**1.15–2.86**
Multivariable analysis (somatic)*	None/mild/moderate	**Ref**	
	Severe	**1.98**	**1.24–3.16**
**Psychological menopausal symptoms**	None/mild/moderate	**Ref**	
	Severe	**1.62**	**1.09–2.40**
Multivariable analysis (psychological)*	None/mild/moderate	**Ref**	
	Severe	**1.76**	**1.17–2.65**
**Urogenital menopausal symptoms**	None/mild/moderate	Ref	
	Severe	1.01	0.67–1.51
Multivariable analysis (urogenital)*	None/mild/moderate	Ref	
	Severe	1.06	0.70–1.61
**Number of severe symptoms**	0/1	Ref	
	2/3	1.48	0.96–2.26
Multivariable analysis (number of severe symptoms)*	0/1	**Ref**	
	2/3	**1.59**	**1.03–2.46**
**Menopausal status**	Premenopausal	Ref	
	Perimenopausal	1.03	0.64–1.66
	Postmenopausal	0.87	0.52–1.45

*adjusted for age and ethnicity

Looking at menopausal symptoms as separate domains, we found an association between suboptimal HIV clinic attendance and severe somatic (AOR 1.98; 95% CI 1.24, 3.16) and psychological (AOR 1.76; 95% CI 1.17, 2.65), but not with severe urogenital symptoms (AOR 1.06; 95% CI 0.70, 1.61). There was also an association between number of domains within which severe symptoms were reported; women experiencing severe symptoms in two or three domains had over 1.5 times the odds of suboptimal HIV clinic attendance when compared to those who reported no severe symptoms or one domain (AOR 1.59; 95% CI 1.03, 2.46, Table 3).

## Discussion

We present one of the largest studies in women living with HIV to date, exploring the association between menopausal status and symptoms, and engagement with HIV care. To the best of our knowledge, this is the first to look specifically at the association between menopausal symptoms and HIV clinic attendance in this group.

We found that women reporting severe menopausal symptoms had over twice the odds of suboptimal adherence to ART. In addition, women reporting severe menopausal symptoms had over 1.5 times the odds of suboptimal HIV clinic attendance compared to those reporting no/mild/moderate symptoms. Taken together, these results suggest that severe menopausal symptoms may negatively impact women’s ability to manage their HIV, further compromising their health and well-being. Suboptimal adherence was also associated with having a detectable viral load, but this association did not reach statistical significance.

We found a clear association between severe menopausal symptoms and adherence to ART. Stratified analyses by individual subdomains (somatic, urogenital and psychological) revealed an association in univariable analyses that were not observed on multivariable analyses. This may be an effect of the sample sizes within the stratified analyses, or may suggest that the effect is a result of the combined experience of a range of menopausal symptoms. Results of the HIV clinic attendance analysis were clearer; both somatic and psychological symptoms were associated with suboptimal HIV clinic attendance, indicating that these two factors may be the predominant drivers of the association between suboptimal clinic attendance and severe menopausal symptoms. Psychological distress has been shown to have an association with clinic attendance in analyses of other populations, (Bowser et al., 2010) indicating that this, in particular, may be an avenue for further investigation.

Although this study is the first in the UK to examine the association between menopausal symptoms and engagement with HIV care, the results of our adherence analysis reflect those of a recent smaller conducted among women with HIV in Vancouver, Canada. Although the results of that study did not reach statistical significance, the analysis of 109 peri- and postmenopausal women found increased odds of suboptimal ART adherence among women suffering from severe menopausal symptoms (Duff et al., 2018). The findings from both studies (in Canada and England) highlight the need for further investigation of the effect of menopausal symptoms on engagement with HIV care in other geographic contexts (where culture and health infrastructure may vary).

There are limitations to this study. The analysis samples were both smaller than the entire PRIME sample, leaving potential for bias if there was a systematic difference between the entire sample and the groups chosen for analysis. This seems unlikely, however, as distributions of every variable were similar across the entire sample and both analysis groups. With regards to menopausal symptoms, there is a question of specificity; are menopausal symptoms acting as a proxy for other (potentially HIV-related) illnesses that may have an effect on engagement with care? Although the experience of menopausal symptoms in women with other comorbidities is likely to be complex, previous validation of the MRS score has indicated that the questions are specific to the menopause (Heinemann et al., 2004). Furthermore, previous analyses of PRIME study data have shown an association between menopausal symptoms (as measured by the MRS) and menopausal status (Tariq S et al., 2017), with an increased likelihood of reporting symptoms in each sub-domain amongst perimenopausal women (which is when you would expect peak symptoms). There is, however, the possibility that psychological symptoms may be driven by factors other than the menopause, indicating a need for further qualitative research on psychological wellbeing and the experience of engagement in HIV care among women of this age group. In addition, it will be important to specifically validate tools such as MRS in women living with HIV, to ensure that these tools are accurately capturing menopausal symptoms in this population.

Data for the PRIME study were collected within a clinical setting, meaning that PRIME participants were engaged with HIV care to at least some degree – further data collection would be required to analyse the relationship between menopausal symptoms and engagement with care among those who are not accessing care at all. Given that the data were collected via questionnaire, there is a risk of social desirability bias with regards to certain questions, particularly those regarding adherence. However, this was avoided as much as possible through both anonymity and the use of validated tools. In addition, it would appear unlikely that women with severe menopausal symptoms would be more likely to misrepresent their adherence than those without, or vice-versa.

The ethnicity of the majority of our sample was black African, which may limit the generalisability of the results to other ethnic and cultural groups. However, this sample is largely representative of the population of women living with HIV within the United Kingdom which is 65% black African (Kirwan PD et al., 2016). Finally, the cross-sectional nature of the study limits our ability to infer causality. Menopausal symptoms may impact engagement with care, but equally, poor adherence to ART may lead to increased HIV-related symptoms (mimicking menopausal symptoms), or indeed worsen menopausal symptoms themselves, and poor clinic attendance may be a sign that women do not engage with any care, leaving menopausal symptoms more likely to remain untreated. This indicates scope for further mixed-methods investigation into the link between engagement with HIV care and clinical HIV outcomes; regardless of the direction of causality, the correlation between menopausal symptoms and engagement in care highlights the important role of clinical and social support to ensure appropriate management of menopausal symptoms and HIV during the menopause transition.

This paper also has numerous strengths. It is the largest investigation to date of the relationship between menopausal symptoms and engagement with HIV care, and validated tools were used to assess both menopausal symptom severity and ART adherence. The sample was broadly representative of women receiving HIV care within the UK; the multicentre PRIME study collected data from National Health Service (NHS) HIV clinics across England, with a questionnaire response rate of 80% (Tariq et al., 2019). As such, this study represents an important contribution to our understanding of the ageing process among women living with HIV, an area that suffers from a significant lack of data, despite the clear implications for clinical practice.

Our analyses highlight an association between severe menopausal symptoms and poor engagement in HIV care (as indicated by ART adherence and HIV clinic attendance) among women with HIV in England aged 45–60. As the population of older women living with HIV continues to increase, it is important that HIV management adapts to the unique challenges that women with HIV face with regards to engagement with HIV care *across* the life-course. We believe that a holistic clinical approach that addresses the multidimensional nature of menopausal symptoms may support midlife women living with HIV in maintaining optimal adherence to ART and clinic attendance. Greater awareness of the role of menopausal symptoms in engagement with care, and the utilisation of integrated clinical pathways that allow primary and secondary care physicians to identify women living with HIV who are experiencing menopausal symptoms, and help them to access appropriate management and support is required. With this in mind we welcome the inclusion of recommendations for proactive assessment of menopausal symptoms in UK HIV guidelines, such as the recent British HIV Association Standards of Care for People Living with HIV (British HIV Association, 2018). It is only by recognising and addressing the changing needs of women living with HIV as they get older that we can ensure we reduce disparities in HIV care and clinical outcomes.
